# Constraining generalized non-local cosmology from Noether symmetries

**DOI:** 10.1140/epjc/s10052-017-5283-x

**Published:** 2017-10-30

**Authors:** Sebastian Bahamonde, Salvatore Capozziello, Konstantinos F. Dialektopoulos

**Affiliations:** 1 Department of Mathematics, University College London, Gower Street, London, WC1E 6BT UK; 2 Dipartimento di Fisica “E. Pancini”, Universitá di Napoli “Federico II”, Naples, Italy; 3 Gran Sasso Science Institute, Via F. Crispi 7, 67100 L’ Aquila, Italy; 4 INFN Sez. di Napoli, Compl. Univ. di Monte S. Angelo, Edificio G, Via Cinthia, 80126 Naples, Italy

## Abstract

We study a generalized non-local theory of gravity which, in specific limits, can become either the curvature non-local or teleparallel non-local theory. Using the Noether symmetry approach, we find that the coupling functions coming from the non-local terms are constrained to be either exponential or linear in form. It is well known that in some non-local theories, a certain kind of exponential non-local couplings is needed in order to achieve a renormalizable theory. In this paper, we explicitly show that this kind of coupling does not need to be introduced by hand, instead, it appears naturally from the symmetries of the Lagrangian in flat Friedmann–Robertson–Walker cosmology. Finally, we find de Sitter and power-law cosmological solutions for different non-local theories. The symmetries for the generalized non-local theory are also found and some cosmological solutions are also achieved using the full theory.

## Introduction

Apart from its remarkable success to interpret cosmological observations, the $\Lambda$-cold dark matter ($\Lambda$CDM) model still lacks according a satisfactory explanation to the issue why the energy density of the cosmological constant is so small if compared to the vacuum energy of the Standard Model (SM) of particle physics. Furthermore, the today observed equivalence, in order of magnitude, of dark matter and dark energy escapes any general explanation a part the introduction of a very strict fine tuning.

Starting from these facts, one cannot consider the cosmological constant fully responsible for the whole anti-gravity dynamics, like the incapability to find a convincing candidate for dark matter, and/or a quantum theory of gravity, many scientists started questioning whether the theory, i.e. general relativity (GR), needed to be changed, in order to explain the accelerating expansion and the large scale structure clustering without the introduction of “ad hoc” cosmological constant and new particles; see, for example, [1–3]. The most usual modifications consist in the introduction of new fields either in the matter sector (e.g. quintessence) or by modifying gravity (e.g. scalar–tensor theories, *f*(*R*), *f*(*T*), etc.). In some sense, the issue is related to adding new matter fields (dark matter, quintessence, etc.) or improving the geometry considering further degrees of freedom of the gravitational field.

Almost a decade ago, a non-local modification of the Einstein–Hilbert (EH) action has been proposed [4], and the new action has the following form:

1 $$\begin{aligned} \mathcal {S}_{\text{ standard-NL }}= & {} \frac{1}{2\kappa }\int \mathrm{d}^{4}x\, \sqrt{-g(x)} \, R(x) \nonumber \\&\times \left[ 1 + f\Big (\left( \square ^{-1}R\right) (x)\Big ) \right] \nonumber \\&+\int \mathrm{d}^{4}x\, \sqrt{-g(x)}\,L_{m}, \end{aligned}$$

where $\kappa =8\pi G$, *R* is the Ricci scalar, *f* is an arbitrary function which depends on the retarded Green function evaluated at the Ricci scalar, $L_{m}$ is any matter Lagrangian and $\square \equiv \partial _{\rho }(e g^{\sigma \rho }\partial _{\sigma })/e$ is the scalar-wave operator, which can be written in terms of the Green function $G(x,x')$ as

2 $$\begin{aligned} (\square ^{-1}F)(x)=\int \mathrm{d}^4x'\, e(x') F(x')G(x,x'). \end{aligned}$$

It is clear that by setting $f(\square ^{-1}R)=0$, the above action is equivalent to the Einstein–Hilbert one plus the matter content. The non-locality is introduced by the inverse of the d’Alembert operator (see [4] for details). Corrections of this kind arise naturally as soon as quantum loop effects are studied and they are also considered as possible solution to the black hole information paradox [5, 6]. Since then, a lot of studies of non-localities have been done in various contexts [7–13]. In Refs. [14–18], non-local quantum gravity is fully discussed putting in evidence results and open issues. From the string theory point of view, in [19] they present some bouncing solutions, in [20] solutions of an expanding Universe with phantom dark energy and in [21] they generate non-Gaussianities during inflation. Emanating from infrared (IR) scales, much progress has also been made. Unification of inflation with late-time acceleration, as well as the dynamics of a local form of the theory, has been studied in [22, 40]. In [23], one shows that non-local gravity models do not alter the GR predictions for gravitationally bound systems, and also they are ghost-free and stable. Finally, in [24–26], one derived a technique to fix the functional form of the function *f* in the action, which is called non-local distortion function. The interested reader is referred to the detailed review by Barvinsky [27], which summarizes the non-local aspects both from the quantum-field theory point of view and from the cosmological one.

Along another track, teleparallel [28] and modified teleparallel theories of gravity [29, 30] have, in the last decade, gained a lot of attention in trying not only to formulate gravity in a gauge invariant way, but also in attempting to interpret the late-time acceleration of the Universe, without invoking any ad hoc cosmological constant. The idea is that gravity, instead of curvature, is mediated only through torsion. This means that the theory is not a geometrical theory anymore, i.e. the trajectories of the particles are not described by geodesic equations, but just by some force equations, since torsion is seen as a force, similar to the Lorentz equation in electrodynamics. The teleparallel equivalent of general relativity (TEGR) is a gauge description of the gravitational interactions and torsion defined through the Weitzenböck connection (instead of the Levi-Civita connection, used by GR, where the equivalence principle is strictly required in order to make geodesic and metric structure to coincide). Hence, in this theory, the manifold is flat but endowed with torsion. The dynamical fields of the theory are the four linearly independent vierbeins and their relation with the metric and the inverse of the metric is given by

3 $$\begin{aligned} g_{\mu \nu } = \eta _{ab}e^a_{\mu } e^b_{\nu }, \quad g^{\mu \nu } = \eta ^{ab}E_a^{\mu } E_b^{\nu }, \end{aligned}$$

where $\eta _{ab}$ is the flat Minkowski metric and $E_a {}^{\mu }$ is the inverse of the tetrads. The action of TEGR is given by

4 $$\begin{aligned} \mathcal {S}_\mathrm{TEGR} =-\frac{1}{2\kappa } \int \mathrm{d}^4 x e\,T +\int \mathrm{d}^{4}x\, e\,L_{m}, \end{aligned}$$

with *e* being $e= \det (e^i{}_{\mu }) = \sqrt{-g}$ and *T* is the torsion scalar, which is given by the contraction

5 $$\begin{aligned} T = S^{\mu \nu }{}_{\rho } T^{\rho }{}_{\mu \nu }, \end{aligned}$$

where

6 $$\begin{aligned}&S_{\rho }{}^{\mu \nu } = \frac{1}{2}\left( K^{\mu \nu }{}_{\rho }+\delta ^{\mu }{}_{\rho }T^{\sigma \nu }{}_{\sigma }-\delta ^{\nu }{}_{\rho }T^{\sigma \mu }{}_{\sigma }\right) , \end{aligned}$$

7 $$\begin{aligned}&K^{\mu \nu }{}_{\rho } = -\frac{1}{2}\left( T^{\mu \nu }{}_{\rho } - T^{\nu \mu }{}_{\rho } - T_{\rho }{}^{\mu \nu } \right) , \end{aligned}$$

8 $$\begin{aligned}&T^{\alpha }{}_{\mu \nu } = \Gamma ^{\alpha }{}_{\mu \nu } - \bar{\Gamma }^{\alpha } {}_{\mu \nu }, \end{aligned}$$

are, respectively, the superpotential, the contorsion tensor, the torsion tensor and $\bar{\Gamma }^{\alpha }{}_{\mu \nu }=E_{a}^{\alpha }\partial _{\mu }e_{\nu }^{a}$ is the Weitzeböck connection. The teleparallelism condition gives the relation of the Ricci scalar with the torsion scalar, that is,

9 $$\begin{aligned} R = -T + \frac{2}{e}\partial _{\mu }(e T^{\mu }) = -T + B. \end{aligned}$$

Hence, we directly see that at the action level, the EH action with the TEGR action differ only by a boundary term and thus the descriptions are equivalent. This is easily generalized to a more complex action as soon as we substitute *T* with an arbitrary function of this, *f*(*T*). This theory can present problems that are non-Lorentz invariant and because a covariant formulation of *f*(*T*) gravity is still not very well accepted since the spin connection is a field without dynamics. Nevertheless, it is always possible to give rise to the correct field equations choosing suitable tetrads (see the review of Ref. [29] for a detailed discussion of advantages and problems related to *f*(*T*) gravity).

The extra degrees of freedom introduced by *f* do not allow us to find an exact relation between *f*(*T*) and *f*(*R*), since now the boundary terms in (9) contribute to the field equations. These kinds of theories and their extensions are of great interest [31–35], since they provide a theoretical interpretation of the accelerating expansion of the Universe and also accommodate the radiation and matter dominated phases of it. In specific cases, one can also find inflationary solutions and avoid the Big Bang singularity with bouncing solutions.

In the teleparallel framework, recently there was proposed a similar kind of non-local gravity based on the torsion scalar *T*. In this theory, the action reads as follows [36]:

10 $$\begin{aligned} \mathcal {S}_{\text{ teleparalell-NL }}= & {} -\frac{1}{2\kappa }\int \mathrm{d}^{4}x\, e(x) \, T(x)\nonumber \\&+\frac{1}{2\kappa }\int \mathrm{d}^{4}x\, e(x) \, T(x)f\Big ((\square ^{-1}T)(x)\Big )\nonumber \\&+\int \mathrm{d}^{4}x\, e(x)\,L_{m}, \end{aligned}$$

where $e=\text {det}(e^{a}_{\mu })=\sqrt{-g}$ and now the function *f* depends on $\square ^{-1}T$. The teleparallel equivalent of GR is recovered if $f(\square ^{-1}T)=0$. It is possible to show [36] that this theory is consistent with the cosmological data by SNe Ia + BAO + CC + $H_0$ observations. From (9), it is straightforward to notice that (1) and (10) correspond to different theories, where *B* is the term connecting them.

Let us now present a generalization of (1) and (10), which we call generalized non-local teleparallel gravity (GNTG). Its action is given by

11 $$\begin{aligned} \mathcal {S}= & {} -\frac{1}{2\kappa }\int \mathrm{d}^{4}x\, e T +\frac{1}{2\kappa }\int \mathrm{d}^{4}x\, e(x) \, (\xi T(x) \nonumber \\&+\,\chi B(x))f\Big ((\square ^{-1}T)(x),(\square ^{-1}B)(x)\Big ) \nonumber \\&+\,\int \mathrm{d}^{4}x\, e(x)\,L_{m}. \end{aligned}$$

Here, *T* is the torsion scalar, *B* is a boundary term and $f(\square ^{-1}T,\square ^{-1}B)$ is now an arbitrary function of the non-local torsion and the non-local boundary terms. The Greek letters $\xi$ and $\chi$ denote coupling constants. It is easily seen that by choosing $\xi = -\chi =-1$ one obtains the standard Ricci scalar. From (2), we directly see that the following relation also holds true:

12 $$\begin{aligned} \square ^{-1}R = -\square ^{-1}T + \square ^{-1}B, \end{aligned}$$

and thus, if $f(\square ^{-1}T,\square ^{-1}B) = f(-\square ^{-1}T + \square ^{-1}B)$, the action takes the well-known form $Rf(\square ^{-1}R)$ given by the action (1). Moreover, non-local teleparalell gravity given by the action (10) is recovered if $\chi =0$ and $f(\square ^{-1}T,\square ^{-1}B)=f(\square ^{-1}T)$. Starting from this theory, we can construct a scalar tensor analog by using Lagrange multipliers and we can constrain the distortion function *f* by the so-called Noether symmetry approach [37]. There are a huge amount of articles in the literature which adopt the Noether symmetry approach to constraining the form of some classes of theories (see for example [1, 31, 38] and the references therein). In this way, one obtains models that, thanks to the existence of Noether symmetries, present integrals of motion that allow one to reduce the dynamics and then, in principle, to find exact solutions. Besides these technical points, the presence of symmetries fixes couplings and potentials with physical meaning [37]. In such a way, the approach can be considered a sort of criterion to “select” physically motivated theories [39]. Details of the approach will be given in Sect. 3.

**Fig. 1:**
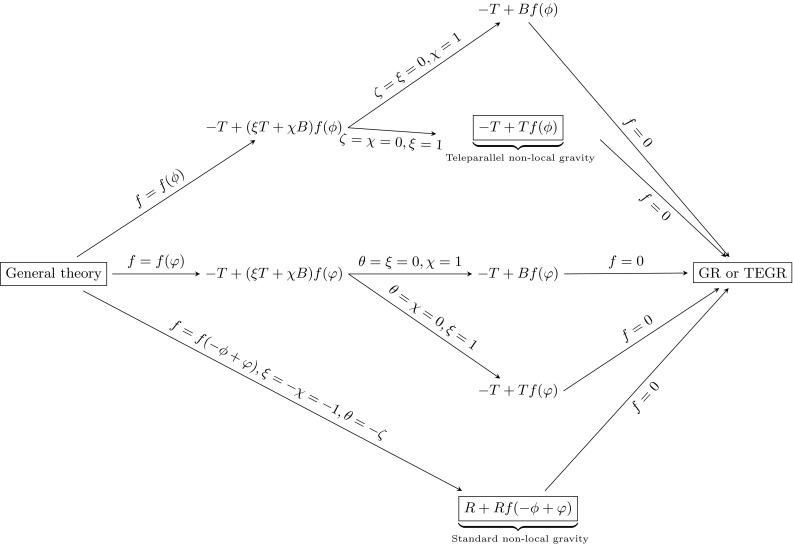
The diagram shows how to recover the different theories of gravity starting from the scalar-field representation of the general theory. Note that $\phi =\square ^{-1}T$ and $\varphi =\square ^{-1}B$ so that $-\phi +\varphi =\square ^{-1}R$. Clearly, the curvature and torsion representations “converge” only for the linear theories in *R*, the GR, and in *T*, the TEGR

The paper is organised as follows: in Sect. 2 we present details of the model, how to construct the action and its scalar–tensor analog with four auxiliary fields. At the end of this section, we present a diagram which shows the different theories that we can construct as subclasses of the general theory. In Sect. 3, we summarize the Noether symmetry approach, which we shall apply to three different cases: (i) the teleparallel non-local case (a coupling like $Tf(\square ^{-1}T)$), in Sect. 4; (ii) the curvature non-local gravity (a coupling like $Rf(\square ^{-1}R)$), in Sect. 5; and (iii) the generalized non-local case (given by the complete action (11)), in Sect. 6. In each case, after the study of the symmetries, we present a set of cosmological solutions. Discussion and conclusions are reported in Sect. 7. Appendix A is devoted to details of the conditions to select the Noether vector. Throughout the paper we adopt the signature $(+,-,-,-)$.

## Generalized non-local cosmology 

Since the field equations for the GNTG theory are very cumbersome, we will rewrite the action (11) in a more suitable way using scalar fields, according to [40]. Specifically, the action can be rewritten introducing four scalar fields $\phi ,\psi ,\theta ,\zeta$ as follows:

13 $$\begin{aligned} \mathcal {S}= & {} -\frac{1}{2\kappa }\int \mathrm{d}^{4}x\, e T + \frac{1}{2\kappa }\int \mathrm{d}^{4}x\, e\left[ \, (\xi T+\chi B)f(\phi ,\varphi )\right. \nonumber \\&\left. +\,\theta (\square \phi - T)+ \zeta (\square \varphi -B)\right] + \int \mathrm{d}^{4}x\, e\,L_{m} ,\nonumber \\= & {} -\frac{1}{2\kappa }\int \mathrm{d}^{4}x\, e T + \frac{1}{2\kappa }\int \mathrm{d}^{4}x\, e\left[ \, (\xi T+\chi B)f(\phi ,\varphi ) \right. \nonumber \\&\left. - \,\partial _{\mu } \theta \partial ^{\mu } \phi - \theta T - \partial _{\mu } \zeta \partial ^{\mu } \varphi -\zeta B \right] + \int \mathrm{d}^{4}x\, e\,L_{m}.\nonumber \\ \end{aligned}$$

By varying this action with respect to $\theta$ and $\zeta$ we get $\phi = \square ^{-1}T$ and $\varphi = \square ^{-1}B$, respectively. In addition, by varying this action with respect to $\phi$ and $\varphi$ we get

14 $$\begin{aligned} \square \theta= & {} (\xi T + \chi B) \frac{\partial f(\phi ,\varphi )}{\partial \phi }, \end{aligned}$$

15 $$\begin{aligned} \square \zeta= & {} (\xi T + \chi B) \frac{\partial f(\phi ,\varphi )}{\partial \varphi }. \end{aligned}$$

In the scalar representation it is not straightforward how to recover the curvature or teleparallel non-local gravity. Let us explicitly recover these theories in the scalar formalism. For example, by setting $\xi = -1 = - \chi$, $f(\phi ,\varphi ) = f(-\phi +\varphi )$, and $\theta =- \zeta$ we obtain a standard form of non-local curvature gravity, namely

16 $$\begin{aligned} \mathcal {S}_{\text{ standard-NL }}= & {} \frac{1}{2\kappa }\int \mathrm{d}^{4}x\, \sqrt{-g} \left[ \,R + R f(\psi )\right. \nonumber \\&\left. -\, \partial _{\mu } \zeta \partial ^{\mu } \psi - \zeta R \right] + \int \mathrm{d}^{4}x\, e\,L_{m},\end{aligned}$$

17 $$\begin{aligned}= & {} \frac{1}{2\kappa }\int \mathrm{d}^{4}x\, \sqrt{-g} \left[ \, R + R f(\square ^{-1}R) \right] \nonumber \\&+\, \int \mathrm{d}^{4}x\, e\,L_{m}, \end{aligned}$$

where $\psi = -\phi + \varphi$. On the other hand, the non-local TEGR is recovered if in the action (13) we choose $\xi = 1,\,\chi =0$, $f(\phi ,\varphi ) = f(\phi )$ and $\zeta =0$. We obtain

18 $$\begin{aligned} \mathcal {S}_{\text{ teleparallel-NL }}= & {} \frac{1}{2\kappa }\int \mathrm{d}^{4}x\, e\left[ \, T\left( f(\phi )-1\right) \right. \nonumber \\&\left. -\, \partial _{\mu } \theta \partial ^{\mu } \phi - \theta T \right] + \int \mathrm{d}^{4}x\, e\,L_{m} \end{aligned}$$

19 $$\begin{aligned}= & {} \frac{1}{2\kappa }\int \mathrm{d}^{4}x\, e\left[ \, T\left( f(\square ^{-1}T)-1\right) \right] \nonumber \\&+\, \int \mathrm{d}^{4}x\, e\,L_{m}. \end{aligned}$$

A more general class of theories, like $-T + (\xi T+\chi B) f(\square ^{-1}T)$ or $-T + (\xi T+\chi B) f(\square ^{-1}B)$ can be obtained by setting $f(\phi ,\varphi ) = f(\phi )$ and $f(\phi ,\varphi ) = f(\varphi )$, respectively. Obviously, in these cases, one can change the values of $\xi$ and $\chi$ to obtain other couplings like

20 $$\begin{aligned} S= & {} \frac{1}{2\kappa }\int \mathrm{d}^{4}x\, e\left[ \, -T + B f(\square ^{-1}T) \right] + \int \mathrm{d}^{4}x\, e\,L_{m}, \end{aligned}$$

21 $$\begin{aligned} S= & {} \frac{1}{2\kappa }\int \mathrm{d}^{4}x\, e\left[ \,-T + T f(\square ^{-1}B) \right] + \int \mathrm{d}^{4}x\, e\,L_{m}, \end{aligned}$$

22 $$\begin{aligned} S= & {} \frac{1}{2\kappa }\int \mathrm{d}^{4}x\, e\left[ -T + B f(\square ^{-1}B) \right] + \int \mathrm{d}^{4}x\, e\,L_{m}. \end{aligned}$$

Figure 1 is a comprehensive diagram representing all the theories that can be recovered from the action (13). Here, we have not considered unnatural couplings like $Rf(\square ^{-1}T)$ or $Tf(\square ^{-1}R)$ because *R* and *T*, *B* are quantities defined in different connections, so mixed terms like $Rf(\square ^{-1}T)$ are badly defined. The above half part of the figure represents different non-local teleparallel theories and the below part of it, the standard curvature counterpart. As is easy to see, only TEGR and GR dynamically coincide while this is not the case for other theories defined by *T*, *R* and *B*. From a fundamental point of view, this fact is extremely relevant because the various representations of gravity can have different dynamical contents. For example, it is well known that *f*(*T*) gravity gives second order field equations, while *f*(*R*) gravity, in metric representation, is fourth order. These facts are strictly related to the dynamical roles of torsion and curvature and their discrimination at fundamental level could constitute important insight in really understanding the nature of the gravitational field (see [29] for a detailed discussion).

By varying the generalized non-local action (13) with respect to the tetrads, we get the following field equations:

23 $$\begin{aligned}&2(1-\xi (f(\phi ,\varphi )-\theta ))\left[ e^{-1}\partial _\mu (e S_{a}{}^{\mu \beta }) \right. \nonumber \\&\quad \left. -\,E_{a}^{\lambda }T^{\rho }_{\mu \lambda }S_{\rho }{}^{\beta \mu }-\frac{1}{4}E^{\beta }_{a}T\right] \nonumber \\&\quad -\,\frac{1}{2}\Big [(\partial ^{\lambda }\theta )(\partial _{\lambda }\phi )E_{a}^{\beta }-(\partial ^{\beta }\theta )(\partial _{a}\phi )-(\partial _{a}\theta )(\partial ^{\beta }\phi )\Big ]\nonumber \\&\quad -\,\frac{1}{2}\Big [(\partial ^{\lambda }\zeta )(\partial _{\lambda }\varphi )E_{a}^{\beta }-(\partial ^{\beta }\zeta )(\partial _{a}\varphi )-(\partial _{a}\zeta )(\partial ^{\beta }\varphi )\Big ] \nonumber \\&\quad +\,2\,\partial _{\mu }\Big [f(\phi ,\varphi )(\xi +\chi )-\theta -\zeta \Big ]E^\rho _a S_{\rho }{}^{\mu \nu }\nonumber \\&\quad + \,\Big (E^{\nu }_{a}\Box -E^\mu _a \nabla ^{\nu }\nabla _{\mu }\Big )(\zeta -\chi f(\phi ,\varphi ))= \kappa \Theta ^\beta _a, \end{aligned}$$

where $\Theta ^\beta _a$ is the general energy-momentum tensor.

Let us now take into account the tetrad $e^{a}_{\beta }=(1,a(t), a(t),a(t)), \,$, which reproduces the flat Friedmann–Robertson–Walker (FRW) metric $ds^2=dt^2-a(t)^2(dx^2+dy^2+dz^2)$. For this geometry, the modified FRW equations are

24 $$\begin{aligned}&3 H^2 (\theta -\xi f+1) = \frac{1}{2} \dot{\zeta } \dot{\varphi }+\frac{1}{2} \dot{\theta }\dot{\phi } \nonumber \\&\quad +\,3 H \big (\dot{\zeta }-\chi \dot{f}\big )+\kappa \rho _m, \end{aligned}$$

25 $$\begin{aligned}&\big (2\dot{H}+3 H^2\big ) (\theta -\xi f+1) = - \frac{1}{2}\dot{\zeta } \dot{\varphi }-\frac{1}{2} \dot{\theta } \dot{\phi } \nonumber \\&\quad -\,\dot{f} (2 H (\xi +2 \chi )+\chi ) +2H(2 \dot{\zeta }+\dot{\theta })+ \ddot{\zeta }-\kappa p_m, \end{aligned}$$

where $\rho _m$ and $p_m$ are the energy density and the pressure of the cosmic fluid, respectively, and dots denote differentiation with respect to the cosmic time. The equations for the scalar fields can be written as

26 $$\begin{aligned}&6 H^2+3 H \dot{\phi }+\ddot{\phi }=0, \end{aligned}$$

27 $$\begin{aligned}&6 (\dot{H}+3 H^2)+3 H \dot{\varphi }+\ddot{\varphi }=0, \end{aligned}$$

28 $$\begin{aligned}&-\,6 H^2 \left( \xi f_{\varphi }+3 \chi f_{\varphi }\right) -6 \dot{H} \chi f_{\varphi }+3 H \dot{\zeta }+ \ddot{\zeta }=0, \end{aligned}$$

29 $$\begin{aligned}&-\,6 H^2 \left( \xi f_{\phi }+3 \chi f_{\phi }\right) -6 \dot{H} \chi f_{\phi }+3 H \dot{\theta }+ \ddot{\theta }=0, \end{aligned}$$

where the sub-indices represent the partial derivative $f_{\phi }=\partial f/\partial \phi$ and $f_{\varphi }=\partial f/\partial \varphi$. In the following section, we will use the Noether symmetry approach to seeking conserved quantities.

## The Noether symmetry approach

Let us use the Noether symmetry approach [37, 41] in order to find symmetries and cosmological solutions for the generalized action (13). For simplicity, hereafter we will study the vacuum case, i.e., $\rho _m=p_m=0$. It can be shown that the torsion scalar and the boundary term in a flat FRW are given by

30 $$\begin{aligned} T=-6H^2, \ \ B=-18H^2-6\dot{H}, \end{aligned}$$

so that the action (13) takes the following form:

31 $$\begin{aligned} \mathcal {S}= & {} 2\pi ^{2}\int a^3\mathrm{d}t\left\{ -6\frac{\dot{a}^2}{a^2}(\xi f(\phi ,\varphi ) -\theta - 1) \right. \nonumber \\&\left. -\,6\Big (2\frac{\dot{a}^2}{a^2} - \frac{\ddot{a}}{a}\Big )(\chi f(\phi ,\varphi ) - \zeta )-\dot{\theta }\dot{\phi }-\dot{\zeta }\dot{\varphi }\right\} . \end{aligned}$$

Considering the procedure in [37], we find that the point-like Lagrangian is given by

32 $$\begin{aligned} \mathcal {L}= & {} 6 a\dot{a}^2 \big (\theta +1-\xi f(\phi ,\varphi )\big )\nonumber \\&+\,6 a^2\dot{a}(\chi \dot{f}(\phi ,\varphi )-\dot{\zeta })- a^3 \dot{\theta }\dot{\phi }- a^3 \dot{\zeta }\dot{\varphi }. \end{aligned}$$

The generator of infinitesimal transformations [41] is given by

33 $$\begin{aligned} X = \lambda (t,x^{\mu })\partial _t + \eta ^i (t,x^{\mu })\partial _i, \end{aligned}$$

where $x^{\mu }=(a,\theta ,\phi ,\varphi ,\zeta )$ and the vector $\eta ^i$ is

34 $$\begin{aligned} \eta ^i (t,x^{\mu })=\Big (\eta ^{a},\eta ^{\theta },\eta ^{\phi },\eta ^{\varphi },\eta ^{\zeta }\Big ). \end{aligned}$$

In general, each function depends on *t* and $x^{\mu }$. If there exists a function $h=h(t,x^{\mu })$ such that

35 $$\begin{aligned} X^{[1]}\mathcal {L}+\mathcal {L}\frac{\mathrm{d}\lambda }{\mathrm{d}t} = \frac{\mathrm{d}h}{\mathrm{d}t}, \end{aligned}$$

where $\mathcal {L}=\mathcal {L}(t,x^{\mu },\dot{x}^{\mu })$ is the Lagrangian of a system and $X^{[1]}$ is the first prolongation of the vector *X* [41], then the Euler–Lagrange equations remain invariant under these transformations. The generator is a Noether symmetry of the system described by $\mathcal {L}$ and the relative integral of motion is given by

36 $$\begin{aligned} I = \lambda \left( \dot{x}^{\mu }\frac{\partial \mathcal {L}}{\partial \dot{x}^{\mu }}-\mathcal {L}\right) - \eta ^i \frac{\partial \mathcal {L}}{\partial \dot{x}^{\mu }} + h. \end{aligned}$$

In the next subsections, we will search for Noether symmetries in specific non-local Lagrangians, starting from the two cases $(Tf(\square ^{-1}T) \,\text {and}\, Rf(\square ^{-1}R))$ and ending with the general action (13). The set of generalized coordinates $x^{\mu } = \{t,a,\theta ,\phi ,\varphi ,\zeta \}$ gives rise to the configuration space $\mathcal {Q} \equiv \{x^{\mu },\mu =1,\ldots ,6\}$ and the tangent space $\mathcal {T}\mathcal {Q} \equiv \{x^{\mu },\dot{x}^{\mu }\}$ of the Lagrangian $\mathcal {L}=\mathcal {L}(t,x^{\mu },\dot{x}^{\mu })$. Clearly, the procedure can be applied to many different models starting from Fig. 1.

## Noether’s symmetries in teleparallel non-local gravity with coupling $Tf(\square ^{-1}T)$

### Finding Noether’s symmetries

Let us first study the case where we recover the teleparallel non-local case studied in [36]. In this case, the torsion scalar *T* is coupled with a non-local function evaluated at the torsion scalar, that is, $f(\square ^{-1}T)=f(\phi )$. For Noether symmetries, we need to consider

37 $$\begin{aligned} f(\phi ,\varphi )=f(\phi ), \quad \chi =0, \quad \xi =1 \,\quad \text {and} \,\quad \zeta = 0, \end{aligned}$$

in the general action (13) and thus the Lagrangian becomes

38 $$\begin{aligned} \mathcal {L} = 6 a \left( - f(\phi ) + \theta + 1\right) \dot{a}^2 - a^3 \dot{\theta } \dot{\phi }. \end{aligned}$$

From Eq. (35), one derives a system of 16 equations for the coefficients of the Noether vector and the functions *h*, *f*. It can be x seen v that the dependence on the coordinates of the Noether vector components is

39 $$\begin{aligned} \lambda (a,\theta ,\phi ,t)= & {} \lambda (t), \end{aligned}$$

40 $$\begin{aligned} \eta _{a}(a,\theta ,\phi ,t)= & {} \eta _{a}(a,\theta ,\phi ,t), \end{aligned}$$

41 $$\begin{aligned} \eta _{\phi }(a,\theta ,\phi ,t)= & {} \eta _{\phi }(a,\phi ,t),\end{aligned}$$

42 $$\begin{aligned} \eta _{\theta }(a,\theta ,\phi ,t)= & {} \eta _{\theta }(a,\theta ,t),\end{aligned}$$

43 $$\begin{aligned} h(a,\theta ,\phi ,t)= & {} h(a,\theta ,\phi ). \end{aligned}$$

The whole system can be straightforwardly derived from the general one in Appendix A (see also [41, 42] for details). Note that we do not need to impose any ansatz to find the symmetries. Hence, the equation for *f* reads

44 $$\begin{aligned} c_1 f'(\phi ) - c_2 f(\phi ) + c_2 - c_3 =0 , \end{aligned}$$

where $c_1,c_2$ and $c_3$ are constants. There are two non-trivial solutions ($f\ne$ constant) to (44) depending on the value of $c_2$, i.e.

45 $$\begin{aligned} f(\phi )= \left\{ \begin{array}{lll} c_7 e^{\frac{c_2 \phi }{c_1}}-\frac{c_3}{c_2}+1, &{}&{} c_{2}\ne 0,\\ c_7 + \frac{c_3 }{c_1}\phi , &{}&{} c_2 = 0, \end{array}\right. \end{aligned}$$

where $c_7$ is another integration constant. From (19), we can notice that for having a TEGR (or GR) background we must have $c_3=c_2$ in the exponential form and $c_7=0$ in the linear form. The Noether vector has the following form:

46 $$\begin{aligned} X = (c_4 + c_5 t) \partial _t -\frac{1}{3}(c_2-c_4)a\partial _a +( c_3+c_2 \theta ) \partial _{\theta } + c_1 \partial _{\phi } , \end{aligned}$$

and the integral of motion is

47 $$\begin{aligned} I= & {} a^3 c_1 \dot{\theta } + a^3 c_2 (\theta +1) \dot{\phi } - a^3 \left( c_4 t+c_5\right) \dot{\theta } \dot{\phi }\nonumber \\&+\left[ 4 a^2 \left( c_2-c_4\right) \dot{a}+6 a \dot{a}^2 \left( c_4 t+c_5\right) \right] \nonumber \\&\times (-f(\phi )+\theta +1)+c_6. \end{aligned}$$

### Cosmological solutions

In the previous subsection we found that the form of the function *f* is constrained to be an exponential or a linear form of the non-local term (45). It can be shown that for the linear form, there are no power-law or de Sitter solutions. Here we will find solutions for the exponential form of the coupling function.

As we pointed out before, it is physically convenient to choose $c_2=c_3$ in order to have a GR (or TEGR) background. Hence, in this section, we will assume this condition for the constants. For the exponential form of the function $f(\phi )$ given by (45), the Lagrangian (38) now takes the form

48 $$\begin{aligned} \mathcal {L} = -6 a\dot{a}^2 \left( c_7 e^{\frac{c_3 \phi }{c_1}}-\theta -1\right) - a^3 \dot{\theta } \dot{\phi }, \end{aligned}$$

so that the Euler–Lagrange equations are given by

49 $$\begin{aligned}&c_1 \left( 4 \dot{H} (c_7 e^{\frac{c_2 \phi }{c_1}}-\theta -1)-\dot{\theta } \dot{\phi }\right) \nonumber \\&\quad +\,H \left( 4 c_2 c_7 \dot{\phi } e^{\frac{c_2 \phi }{c_1}}-4 c_1 \dot{\theta }\right) \nonumber \\&\quad +\,6 c_1 H^2 \left( c_7 e^{\frac{c_2 \phi }{c_1}}-\theta -1\right) = 0 , \end{aligned}$$

50 $$\begin{aligned}&6 H^2+3 H \dot{\phi }+\ddot{\phi } = 0 , \end{aligned}$$

51 $$\begin{aligned}&-\frac{6 c_2 c_7 }{c_1}H^2 e^{\frac{c_2 \phi }{c_1}}+\ddot{\theta }+3 H \dot{\theta } = 0 , \end{aligned}$$

52 $$\begin{aligned}&6H^2 \left( - c_7 e^{\frac{c_2 \phi }{c_1}}+ \theta +1\right) - \dot{\theta } \dot{\phi }+6 \theta H^2 = 0 , \end{aligned}$$

for $a,\theta ,\phi$ and the energy equation, respectively. If we consider de Sitter solution for the scale factor,

$$\begin{aligned} a(t) = e^{H_0 t} \Rightarrow H(t) = H_0, \end{aligned}$$

we immediately find from (50) that

53 $$\begin{aligned} \phi (t) = -2 H_0 t-\frac{\phi _1 e^{-3 H_0 t}}{3 H_0} + \phi _2 . \end{aligned}$$

For the sake of simplicity, we will choose $\phi _1 =\phi _2= 0$ otherwise Eq. (51) cannot be integrated easily. By this assumption, we directly find that

54 $$\begin{aligned} \theta (t) = e^{-3 H_0 t} \left( -c_7 (3 H_0 t+1)-\frac{\theta _1}{3 H_0}\right) +\theta _2, \end{aligned}$$

where $\theta _1$ and $\theta _2$ are integration constants and we needed to choose the branch $c_1=2c_2/3$, otherwise Eq. (49) cannot be satisfied. Hence, from (49) we directly see that $\theta _2=-1$, giving us the following cosmological solution:

55 $$\begin{aligned} a(t)= & {} e^{H_0 t} ,\quad \phi (t) = -2 H_0 t , \quad \theta (t)\nonumber \\= & {} e^{-3 H_0 t} \left( -c_7 (3 H_0 t+1)-\frac{\theta _1}{3 H_0}\right) -1, \end{aligned}$$

and

56 $$\begin{aligned} f(\phi ) = c_7 e^{-3 H_0 t}. \end{aligned}$$

If we consider that the scale factor behaves as a power-law $a(t)=a_0t^p$, where *p* is a constant, from (50) we directly find that

57 $$\begin{aligned} \phi (t)=\frac{6 p^2 \log (t-3 p t)}{1-3 p}+\frac{\phi _1}{1-3p} t^{1-3 p}+\phi _0, \end{aligned}$$

where $\phi _1$ and $\phi _0$ are integration constants that for simplicity (as we did before) we will assume that are zero, otherwise (51) cannot be integrated directly. By doing this, we find

58 $$\begin{aligned} \theta (t)= & {} \frac{c_1 t^{1-3 p}}{1-3 p}+c_2 \nonumber \\&+\,\frac{c_7 (3 p-1) (c_1-3 c_1 p) }{c_1 (1-3 p)^2-6 c_2 p^2}(t-3 p t)^{\frac{6c_2 p^2}{c_1-3 c_1 p}}, \end{aligned}$$

where $\theta _0$ and $\theta _1$ are integration constants and we have assumed that $c_1\ne \tfrac{6 c_2 p^2}{(3 p-1)^2}$ and $p\ne 1/3$ since there are not solutions for these other two branches. By replacing this solution in (49) we get $c_2 = \frac{c_1 (2 - 9 p + 9 p^2)}{6 p^2}$ and $\theta _1=-1$, yielding the following solution:

59 $$\begin{aligned}&\phi (t)=\frac{6 p^2 \log (t-3 p t)}{1-3 p}, \nonumber \\&\theta (t)=c_7 (1-3p)^{3-3p} t^{2-3p}+\frac{\theta _0 t^{1-3 p}}{1-3 p}-1, \nonumber \\&a(t)=a_0t^p, \nonumber \\&f(\phi )=c_7e^{\frac{\left( 9 p^2-9 p+2\right) \phi }{6 p^2}}. \end{aligned}$$

Note that the energy condition (52) is satisfied and $p=1/3$ is not a solution.

## Noether’s symmetries in curvature non-local gravity with coupling $Rf(\square ^{-1}R)$

### Finding Noether’s symmetries

Let us find now Noether’s symmetries for the case where curvature non-local gravity is considered. We assume that the coupling $Rf(\square ^{-1}R)$ is present in the action. To recover this case, we must set

60 $$\begin{aligned}&f(\phi ,\varphi ) = f(-\phi +\varphi )=f(\psi ),\quad \nonumber \\&\chi =1, \quad \xi =-1,\quad \theta = - \zeta . \end{aligned}$$

In this way, the Lagrangian (13) reads as follows:

61 $$\begin{aligned} \mathcal {L} = 6 a\dot{a}^2 (f(\psi )+\theta + 1) +6 a^2 \dot{a}(f'(\psi )\dot{\psi }+ \dot{\theta }) + a^3 \dot{\theta } \dot{\psi }, \end{aligned}$$

and Noether’s condition Eq. (35), gives a system of 18 differential equations. Also this is a special case of that presented in Appendix A. The result is

62 $$\begin{aligned} \lambda (a,\theta ,\psi ,t) = \lambda (t)\, \quad \text {and} \quad h(a,\theta ,\psi ,t) = h(a,\theta ,\psi ), \end{aligned}$$

and the system reduces to nine equations. However, the full system is still difficult to solve without any assumption. A simple assumption is choosing $h(a,\theta ,\psi )$ = constant. The last two equations of Noether condition for $f(\psi )$ are

63 $$\begin{aligned}&2 c_2 f'(\psi )+c_1 f(\psi )+c_1-c_3 = 0, \end{aligned}$$

64 $$\begin{aligned}&2 c_2 f''(\psi )+c_1 f'(\psi ) = 0 , \end{aligned}$$

and the Noether vector as a result is found to be

65 $$\begin{aligned} X= & {} (c_5 + c_4 t)\partial _t + \frac{1}{3} a (c_4 - c_1)\partial _{a} \nonumber \\&+\,(c_3 + c_1 \theta )\partial _{\theta } -2 c_2 \partial _{\psi } . \end{aligned}$$

Equations (63) and (64) are easily solved and the form of *f* is

66 $$\begin{aligned} f(\psi )= \left\{ \begin{array}{lll} -1+\frac{c_3}{c_1} + c_6 e^{-\frac{c_1}{c_2} \psi } ,&{}&{} c_1 \ne 0,\\ c_6 + \frac{c_3 }{2 c_2}\psi , &{}&{} c_1 = 0. \end{array}\right. \end{aligned}$$

Again, the form of the function is either exponential or linear in $\psi =\square ^{-1}R$. This result is very interesting since, without assumptions apart from $h=\mathrm{const.}$, the symmetries give the same kind of couplings for both teleparallel and curvature non-local theories. These two couplings can be particularly relevant to get a renormalizable theory of gravity. As discussed in [43], the form of the coupling is extremely important to achieve a regular theory. In particular, the exponential coupling plays an important role in calculations. Here, the symmetry itself is imposing this kind of coupling. In other words, it is not put in by hand but is related to a fundamental principle, i.e. the existence of the Noether symmetry.

### Cosmological solutions

It is well known [40] that non-local theories with exponential coupling, i.e. $R(1+ e^{\alpha \square ^{-1}R})$, have both de Sitter and power-law solutions. In this section, we will verify that the Lagrangian (61) with the coupling (66), given by the symmetry, i.e.

67 $$\begin{aligned} \mathcal {L}= & {} 6 a \left( \frac{c_3}{c_1} + \theta \right) \dot{a}^2 + 3 c_6 a e^{-\frac{c_1}{2 c_2} \psi } \left( 2 \dot{a}^2 - \frac{c_1}{c_2} a \dot{a}\dot{\psi }\right) \nonumber \\&+\, 6 a^2 \dot{a} \dot{\theta } + a^3 \dot{\theta } \dot{\psi }, \end{aligned}$$

gives rise to these solutions. In order to recover the GR background, we will assume that $c_3=c_1$.

Let us start from the de Sitter case, where $a(t) = e^{H_0 t}$. The Euler–Lagrange equations for $a, \psi , \theta$ and the energy equation, read, respectively,

68 $$\begin{aligned}&c_1^2 c_6\dot{\psi }^2+8 c_2^2 \dot{H} \left( \theta e^{\frac{c_1 \psi }{2 c_2}}+e^{\frac{c_1 \psi }{2 c_2}}+c_6\right) \nonumber \\&\quad +\,12 c_2^2 H^2 \left( \theta e^{\frac{c_1 \psi }{2 c_2}}+e^{\frac{c_1 \psi }{2 c_2}}+c_6\right) \nonumber \\&\quad +\,4 c_2^2 \ddot{\theta } e^{\frac{c_1 \psi }{2 c_2}}-2 c_2^2 \dot{\theta }\dot{\psi } e^{\frac{c_1 \psi }{2 c_2}}\nonumber \\&\quad +\,4 c_2 H \left( 2 c_2 \dot{\theta } e^{\frac{c_1 \psi }{2 c_2}}-c_1 c_6\dot{\psi }\right) -2 c_1 c_2 c_6 \ddot{\psi }= 0 , \end{aligned}$$

69 $$\begin{aligned}&3 c_1 c_6 \dot{H} e^{-\frac{c_1 \psi }{2 c_2}}+6 c_1 c_6 H^2 e^{-\frac{c_1 \psi }{2 c_2}}-3 c_2 H \dot{\theta }-c_2 \ddot{\theta } = 0 , \nonumber \\\end{aligned}$$

70 $$\begin{aligned}&6 \dot{H}+3 H\dot{\psi }+12 H^2+\ddot{\psi }= 0 , \end{aligned}$$

71 $$\begin{aligned}&H \left( 6 \dot{\theta }-\frac{3 c_1 c_6\dot{\psi } e^{-\frac{c_1 \psi }{2 c_2}}}{c_2}\right) \nonumber \\&\quad +\,6 H^2 \left( c_6 e^{-\frac{c_1 \psi }{2 c_2}}+\theta +1\right) +\dot{\theta }\dot{\psi } =0 . \end{aligned}$$

Equation (70) gives

72 $$\begin{aligned} \psi (t) = -4 H_0 t -\frac{\psi _1 e^{-3 H_0 t}}{3 H_0} + \psi _2, \end{aligned}$$

where $\psi _1$ and $\psi _0$ are integration constants. For simplicity, to find analytical solutions, we set $\psi _1=\psi _0 =0$. Then from Eq. (69) we find

73 $$\begin{aligned} \theta (t) = \frac{3 c_2 c_6 }{2 c_1+3 c_2} e^{\frac{4 c_1 H_0 t - c_1 \psi _2}{2 c_2}} -\frac{\theta _1 }{3 H_0} e^{-3 H_0 t}+ \theta _2, \end{aligned}$$

and, in order to satisfy the other two Eqs. (68) and (71), we set $$\theta _2 = - 1$$ and $$ c_2 = -c_1$$. Finally, the following de Sitter solution:

74 $$\begin{aligned} a(t)= & {} e^{H_0 t} , \quad \psi (t) = - 4 H_0 t + \psi _2 , \quad \nonumber \\ \theta (t)= & {} 3 c_6 e^{\frac{\psi _2}{2}-2 H_0 t}-\frac{\theta _1}{3 H_0} e^{-3 H_0 t}-1, \end{aligned}$$

is recovered and

75 $$\begin{aligned} f(\psi ) = c_6 e^{\psi /2}. \end{aligned}$$

In the same spirit, if we assume that the scale factor with a power-law behavior as $$a(t) = a_0t^p $$, the system (68)–(71) yields the following solution:

76 $$\begin{aligned}&a(t) =a_0 t^p , \quad \psi (t) = \frac{6 p (1-2 p)}{3 p-1} \ln (t) , \nonumber \\&\quad \theta (t) = \frac{c_6 (3 p-1)}{(p-1) }t^{-2 p}-1 , \quad f(\psi (t)) = c_6 e^{\frac{\psi (1-3p)}{3(1-2p)}}.\nonumber \\ \end{aligned}$$

This solution is valid for $p\ne 1/3$. Now, if one considers the linear form of $f(\psi )=c_6+\frac{c_3}{2c_2}\psi$, it is also possible to find power-law solutions but only for $p=1/2$, which corresponds to radiation. The non-trivial solution for this particular case is given by

77 $$\begin{aligned} \theta (t)= & {} \theta _0,\quad a(t)=a_0 t^{1/2}, \quad \nonumber \\ \psi (t)= & {} -\frac{2 c_2 (\theta _0+2)}{c_3}-2\psi _1 t^{-1/2}, \quad \nonumber \\ f(\psi )= & {} \frac{c_3 \psi }{2 c_2}+c_6, \end{aligned}$$

where $\theta _0$ and $\psi _1$ are constants.

## Noether’s symmetries in the general case

### Finding Noether’s symmetries

Let us consider now the generalized non-local action involving both teleparallel and curvature non-local contributions. The Lagrangian is

78 $$\begin{aligned} \mathcal {L}= & {} 6 \chi a^2 \dot{a} \dot{\phi } f_{\phi }(\phi ,\varphi )+6 \chi a^2 \dot{a} \dot{\varphi } f_{\varphi }(\phi ,\varphi )-6 \xi a \dot{a}^2 f(\phi ,\varphi ) \nonumber \\&-\,6 a^2 \dot{a} \dot{\zeta } + 6 a \theta \dot{a}^2 + 6 a \dot{a}^2 - a^3 \dot{\zeta } \dot{\varphi } - a^3 \dot{\theta } \dot{\phi }, \end{aligned}$$

from which we can derive several interesting theories as shown in the diagram; see Fig. 1. The Noether condition (35) gives a system of 43 (non-independent) equations for the Noether vector components

79 $$\begin{aligned}&\lambda (a,\theta ,\phi ,\varphi ,\zeta ,t),\,\,\eta _{a}(a,\theta ,\phi ,\varphi ,\zeta ,t),\,\,\nonumber \\&\quad \eta _{\phi }(a,\theta ,\phi ,\varphi ,\zeta ,t),\,\,\eta _{\varphi }(a,\theta ,\phi ,\varphi ,\zeta ,t),\,\,\nonumber \\&\quad \eta _{\theta }(a,\theta ,\phi ,\varphi ,\zeta ,t),\,\,\eta _{\zeta }(a,\theta ,\phi ,\varphi ,\zeta ,t), \end{aligned}$$

and the functions

80 $$\begin{aligned} h(a,\theta ,\phi ,\varphi ,\zeta ,t),\,\,f(\phi ,\varphi ). \end{aligned}$$

We can see immediately, from the system, that

81 $$\begin{aligned} \lambda (a,\theta ,\phi ,\varphi ,\zeta ,t)= & {} \lambda (t), \end{aligned}$$

82 $$\begin{aligned} \eta _{\phi }(a,\theta ,\phi ,\varphi ,\zeta ,t)= & {} \eta _{\phi }(a,\phi ,\varphi ,\zeta ,t), \end{aligned}$$

83 $$\begin{aligned} h(a,\theta ,\phi ,\varphi ,\zeta ,t)= & {} h(a,\theta ,\phi ,\varphi ,\zeta ). \end{aligned}$$

The system now reduces to 19 equations, which cannot easily be solved (see Appendix A for details). Hence, as we did in the previous sections, we assume that $h(a,\theta ,\phi ,\varphi ,\zeta ) =\text{ constant } = h$ and after some calculations we end up with the following three equations for $f(\phi ,\varphi )$:

84 $$\begin{aligned}&-f_{\varphi }(\phi ,\varphi ) \left( c_7 \xi \varphi +c_6 \xi +c_8 \xi -6 c_7 \chi \right) \nonumber \\&\quad +\,f_{\phi }(\phi ,\varphi ) \left( -c_5 \xi \varphi -c_4 \xi +6 c_5 \chi \right) -6 c_7 \chi \phi f_{\varphi \phi }(\phi ,\varphi )- \nonumber \\&\quad -\,6 c_5 \chi \phi f_{\phi \phi }(\phi ,\varphi ) \nonumber \\&\quad +\,c_3 \xi f(\phi ,\varphi )-c_3+c_{10}-c_{12} = 0, \end{aligned}$$

85 $$\begin{aligned}&6 \left( c_7-c_3\right) \chi f_{\varphi }(\phi ,\varphi )+6 \chi \left( c_7 \varphi +c_6+c_8\right) f_{\varphi \varphi }(\phi ,\varphi )\nonumber \\&\quad +\,6 c_5 \chi f_{\phi }(\phi ,\varphi ) \nonumber \\&\quad +\,6 \chi \left( c_5 \varphi -c_7 \phi +c_4\right) f_{\varphi \phi }(\phi ,\varphi )\nonumber \\&\quad -\,6 c_5 \chi \phi f_{\phi \phi }(\phi ,\varphi )-c_{12} = 0 , \end{aligned}$$

86 $$\begin{aligned}&-\left( c_5 \xi +c_3 \chi \right) f_{\phi }(\phi ,\varphi )-c_7 \xi f_{\varphi }(\phi ,\varphi )-6 c_7 \chi f_{\varphi \varphi }(\phi ,\varphi )\nonumber \\&\quad +\,\chi \left( c_7 \varphi -6 c_5+c_6+c_8\right) f_{\varphi \phi }(\phi ,\varphi )\nonumber \\&\quad +\,\chi \left( c_5 \varphi +c_4\right) f_{\phi \phi }(\phi ,\varphi ) = 0 , \end{aligned}$$

where all the *c* are constants coming from the coefficients of the Noether vector. System (84)–(86) can easily be integrated but, depending on the vanishing or not of some constants, different solutions can be derived. Specifically, we obtain seven different symmetries described below. The Noether vectors and the function *f* take the following forms.

1.
  - For $c_7 \ne 0$ and $c_3 \ne 0, c_4 \ne \frac{c_5}{c_7}(c_6+c_9)$, we have
    87 $$\begin{aligned} X= & {} (c_1 t + c_2)\partial _t + \frac{1}{3}(c_1 - c_3)a \partial _{a} \nonumber \\&+\, (c_4 + c_5(6 \ln a + \psi )) \partial _{\phi }\nonumber \\&+ \,(c_6 + c_7 (6 \ln a + \varphi )+ c_9) \partial _{\varphi } + c_3 \theta \partial _{\theta } \nonumber \\&+\, ((c_3 - c_7) \zeta - c_5 \theta + c_8 )\partial _\zeta \end{aligned}$$
    and
    88 $$\begin{aligned}&f(\phi ,\varphi ) = \frac{1}{\xi } + \frac{c_{11} \left( c_5 c_6-c_4 c_7+c_5 c_9\right) }{c_3}\nonumber \\&\quad \times \,\exp \left( {\frac{c_3}{c_5 c_6-c_4 c_7+c_5 c_9} \left( c_5 \varphi -c_7 \phi \right) } \right) . \end{aligned}$$
  - For $c_7 \ne 0$ and $c_3 = 0, c_4 = \frac{c_5}{c_7}(c_6+c_9)$, we have
    89 $$\begin{aligned} X= & {} (c_1 t + c_2)\partial _t + \frac{c_1}{3} a \partial _{a} \nonumber \\&+\, (c_4 + c_5(6 \ln a + \varphi )) \partial _{\phi }\nonumber \\&+\, (c_6 + c_7 (6 \ln a + \varphi )+ c_9) \partial _{\varphi }\nonumber \\&+\, (c_8-c_7 \zeta -c_5 \theta ) \partial _{\zeta } \end{aligned}$$
    and
    90 $$\begin{aligned} f(\phi ,\varphi ) = c_{11} + F(-c_7 \phi + c_5 \varphi ). \end{aligned}$$
2.
  -
    i. For $$c_7 = 0 $$ and $$c_5 \ne 0$$ and $$c_3 \ne 0 , c_5 \ne - c_6$$, we have
      91 $$\begin{aligned} X= & {} (c_1 t + c_2)\partial _t + \frac{1}{3}(c_1 - c_3)a \partial _{a} \nonumber \\&+\, (c_4 + c_5(6 \ln a + \varphi )) \partial _{\phi } \nonumber \\&+\, (c_6 + c_9) \partial _{\varphi } + (c_{10}+c_3 \theta )\partial _{\theta }\nonumber \\&+\, (c_3 \zeta - c_5 \theta + c_8 )\partial _\zeta \end{aligned}$$
      and
      92 $$\begin{aligned} f(\phi ,\varphi ) = \frac{c_3-c_{10}}{\xi c_3 } + c_{11} e^{\frac{c_3}{c_6 c_9} \varphi }. \end{aligned}$$
    ii. For $$c_7 = 0 $$ and $$c_5 \ne 0$$ and $$c_3 = 0 , c_5 = - c_6$$, we have
      93 $$\begin{aligned} X= & {} (c_1 t + c_2)\partial _t + \frac{c_1}{3}a \partial _{a} \nonumber \\&+\, (c_4 + c_5(6 \ln a + \varphi )) \partial _{\phi } \nonumber \\&+\, (c_8 - c_5 \theta )\partial _\zeta \end{aligned}$$
      and
      94 $$\begin{aligned} f(\phi ,\varphi ) = c_{11} + F (\varphi ). \end{aligned}$$
  -
    i. For $$c_7 = 0 $$ and $$c_5 = 0$$ and $$c_3 \ne 0 , c_4 \ne 0$$, we have
      95 $$\begin{aligned} X= & {} (c_1 t + c_2)\partial _t + \frac{1}{3}(c_1 - c_3)a \partial _{a} \nonumber \\&+\, c_4\partial _{\phi } + (c_6 + c_9) \partial _{\varphi } + (c_{10}+c_3 \theta )\partial _{\theta }\nonumber \\&+\, (c_8 + c_3 \zeta )\partial _\zeta \end{aligned}$$
      and
      96 $$\begin{aligned} f(\phi ,\varphi ) = \frac{c_3-c_{10}}{\xi c_3 } + F\left( -\frac{c_6+c_9}{c_4}\phi +\varphi \right) e^{\frac{c_3}{c_4} \phi }. \end{aligned}$$
    ii.
      A. For $$c_7 = 0 $$ and $$c_5 = 0$$ and $$c_3 = 0 , c_4 = 0$$ and $$c_6 \ne -c_7$$, we have
        97 $$\begin{aligned} X=(c_1 t + c_2)\partial _t + \frac{c_1}{3}a \partial _{a} + (c_6 + c_9) \partial _{\varphi } + c_{10}\partial _{\theta } + c_8\partial _\zeta \end{aligned}$$
        and
        98 $$\begin{aligned} f(\phi ,\varphi ) = \frac{c_{10}}{(c_6+c_9)\xi }\varphi + F(\phi ). \end{aligned}$$
      B. For $$c_7 = 0 $$ and $$c_5 = 0$$ and $$c_3 = 0 , c_4 = 0$$ and $$c_6 = -c_7$$, we have
        99 $$\begin{aligned} X=(c_1 t + c_2)\partial _t + \frac{c_1}{3}a \partial _{a} + c_8 \partial _\zeta , \end{aligned}$$
        and the equations are satisfied for any *f*.

Clearly, each of these symmetries specify a different Lagrangian and thus a different dynamics. As discussed in Appendix A, the fact that several symmetries exist for the same symmetry condition (35) is due to the fact that such a condition consists in a system of non-linear partial differential equations which have no unique general solution.

### Cosmological solutions

Let us now find cosmological solutions for the generalized Lagrangian (78). In principle, it is possible to find cosmological solutions for each of the above cases depending on the coupling functions. Due to the physical importance of the exponential couplings, we will present cosmological solutions for the coupling function given by (88). However, the procedure for the other cases is the same.

In the case (88), we have the constraint given by the integration constants, that is, $c_7 \ne 0 , c_3 \ne 0, c_4 \ne \frac{c_5}{c_7}(c_6+c_9)$. Hence, the Euler–Lagrange equations obtained by (78), together with the energy condition, give a system of six differential equations for $a(t),\phi (t),\varphi (t),\theta (t)\,\text {and}\,\zeta (t)$.

Assuming that the scale factor of the Universe behaves as de Sitter, $a(t)= e^{H_0 t}$, it is possible to find different kinds of solutions depending on different cases for the constants. In all of these cases, the final cosmological solutions are almost the same. A general solution that one can easily find is

100 $$\begin{aligned} a(t)= & {} e^{H_0 t}, \phi (t) = -2 H_0 t, \theta (t) = \frac{1}{3} e^{-3 H_{0} t} \nonumber \\&\times \left( -\frac{18 c_{11} c_7 \chi ^2 (c_7-3 c_5)^2 \exp \left( \frac{H_{0} t (3 c_5-2 c_7) (\xi +3 \chi )}{\chi (3 c_5-c_7)}\right) }{(3 c_5-2 c_7) (3 c_5 \xi -2 c_7 \xi -3 c_7 \chi )}-\frac{\theta _{1}}{H_{0}}\right) , \nonumber \\\end{aligned}$$

101 $$\begin{aligned} \varphi (t)= & {} -6 H_0 t , \zeta (t) = \frac{1}{3} e^{-3 H_{0} t}\nonumber \\&\times \left( \frac{18 c_{11} c_5 \chi ^2 (c_7-3 c_5)^2 \exp \left( \frac{H_{0} t (3 c_5-2 c_7) (\xi +3 \chi )}{\chi (3 c_5-c_7)}\right) }{(3 c_5-2 c_7) (3 c_5 \xi -2 c_7 \xi -3 c_7 \chi )}-\frac{\zeta _{1}}{H_{0}}\right) \nonumber \\&+\zeta _{0}, \end{aligned}$$

and the coupling function *f* becomes

102 $$\begin{aligned} f(\phi ,\varphi )= & {} \frac{1}{\xi }-\frac{2 c_{11} \chi (c_7-3 c_5)^2 }{3 c_5 \xi -2 c_7 \xi -3 c_7 \chi }\nonumber \\&\times \exp \left( -\frac{(3 c_5 \xi -2 c_7 \xi -3 c_7 \chi ) (c_5 \varphi -c_7 \phi )}{2 \chi (c_7-3 c_5)^2}\right) ,\nonumber \\ \end{aligned}$$

where $\theta _1,\zeta _1$ and $\zeta _2$ are integration constants and we need to set ${\displaystyle c_3=-\frac{(3 c_5 \xi -2 c_7 \xi -3 c_7 \chi ) (-c_4 c_7+c_5 c_6+c_5 c_9)}{2 \chi (3 c_5-c_7)^2}}$. Apart from de Sitter solutions, the system admits also power-law solutions. For example, by setting ${\displaystyle c_3=\frac{\left( 9 p^2-9 p+2\right) (-c_4 c_7+c_5 c_6+c_5 c_9)}{6 p (3 c_5 p-c_5-c_7 p)}}$, we get the following solutions:

103 $$\begin{aligned}&a(t)=t^p,\phi (t)=\frac{6 p^2 \ln (t-3 p t)}{1-3 p},\varphi (t)=-6 p \ln t,\nonumber \\&\theta (t)=\frac{6 c_{11} c_7 p t^{2-3 p} (1-3 p)^{\frac{c_7 (2-3 p) p}{-3 c_5 p+c_5+c_7 p}} (p (\xi +3 \chi )-\chi )}{3 p-2}\nonumber \\&\qquad \quad \quad +\,\frac{\theta _{1} t^{1-3 p}}{1-3 p}, \end{aligned}$$

104 $$\begin{aligned}&\zeta (t)-\frac{6 c_{11} c_5 p t^{2-3 p} (1-3 p)^{-\frac{c_7 p (3 p-2)}{-3 c_5 p+c_5+c_7 p}} (p (\xi +3 \chi )-\chi )}{3 p-2}\nonumber \\&\quad +\,\zeta _{0}+\frac{\zeta _{1} t^{1-3 p}}{1-3 p}, \end{aligned}$$

and the coupling function *f* becomes

105 $$\begin{aligned} f(\phi ,\varphi )= & {} \frac{1}{\xi }-\frac{6 c_{11} p (-3 c_5 p+c_5+c_7 p) }{9 p^2-9 p+2}\nonumber \\&\times \, \exp \left( -\frac{\left( 9 p^2-9 p+2\right) (c_5 \varphi -c_7 \phi )}{6 p (-3 c_5 p+c_5+c_7 p)}\right) .\nonumber \\ \end{aligned}$$

The above procedure can be iterated for all the above couplings. We stress again the important fact that such couplings are not arbitrarily given but result from the existence of the symmetries.

## Discussion and conclusions

Motivated by an increasing amount of studies related to non-local theories, here we proposed a new generalized non-local theory of gravity including curvature and teleparallel terms. These kinds of theories were introduced motivated by loop quantum effects and they have attracted a lot of interest since some of them are renormalizable [14]. In suitable limits, the general action that we proposed can represent either curvature non-local theories with $Rf(\square ^{-1}R)$ based on [4] or teleparallel non-local theories $Tf(\square ^{-1}T)$ based on [36]. Since the theory is highly non-linear, it is possible to introduce four auxiliary scalar fields in order to rewrite the action in an easier way. Then, for a flat FRW cosmology, using the Noether symmetry approach, the coupling functions can be selected directly from the symmetries for the various models derived from the general theory. It is obvious that the theory (11) can give several models, depending on the values of the constants $\xi$ and $\chi$ and on the form of the distortion function. We prove that, in most physically interesting cases, the only forms of the distortion function selected by the Noether symmetries are the exponential and the linear ones. According to Refs. [22, 40], this is an important result, because, up to now, these kinds of couplings were chosen by hand in order to find cosmological solutions, while, in our case, they result from a first principle. In addition, there is a specific class of exponentials non-local gravity models which are renormalizable [16, 43]. This means that the Noether symmetries dictate the form of the action and one may choose an exponential form for the distortion function. As discussed in [39], the existence of Noether symmetries is a selection criterion for physically motivated models. Finally, from models selected by symmetries, it is easy to find cosmological solutions like de Sitter and power-law ones. The integrability of the dynamics is guaranteed by the existence of first integrals. In forthcoming studies, the cosmological analysis will be improved in view of the observational data.

## A. The existence conditions for Noether’s symmetry

The vector equation (35),

A1 $$\begin{aligned} X^{[1]}\mathcal {L}+\mathcal {L}\frac{\mathrm{d}\lambda }{\mathrm{d}t} = \frac{\mathrm{d}h}{\mathrm{d}t}, \end{aligned}$$

which we rewrite here for simplicity, gives the existence conditions for the Noether symmetries. This means that the Noether vector components $\eta ^i$ and the functions $\lambda$ and *h* have to be selected. If some of these functions are different from zero, the symmetry exists and, vice versa, selects the class of possible Lagrangians $\mathcal {L}$ compatible with it. These conditions constitute a system of partial differential equations derived by equating to zero the coefficients of time derivatives in Eq. (35). The result of this system is the non-trivial functions $\eta ^i$, $\lambda$, and *h*. The number of differential equations depends on the dimension of the configuration space ${\mathcal Q}$. For a detailed discussion of the method, see [37, 41].

In the present case, considering the general Lagrangian (78), the full Noether conditions are 43 differential equations. However, there are 24 differential equations that can be easily solved leaving us with the functions

A2 $$\begin{aligned}&\lambda =\lambda (t),\quad h=h(a,\theta ,\phi ,\psi ,\zeta ),\quad \nonumber \\&\quad \eta _{\phi }=\eta _{\phi }(a,\phi ,\psi ,\zeta ,t). \end{aligned}$$

The remaining 19 differential equations are the following:

A3 $$\begin{aligned}&h_{,\theta }+a^3\eta _{\phi ,t}=0, \end{aligned}$$

A4 $$\begin{aligned}&h_{,\psi }+a^2 \left( a \eta _{\zeta ,t}-6 \chi f_{,\psi } \eta _{a,t}\right) =0, \end{aligned}$$

A5 $$\begin{aligned}&h_{,\phi }+a^2 \left( a \eta _{\theta ,t}-6 \chi f_{,\phi }\eta _{a,t} \right) =0, \end{aligned}$$

A6 $$\begin{aligned}&h_{,\zeta }+a^2 \left( 6\eta _{a,t} +a\eta _{\psi ,t} \right) =0, \end{aligned}$$

A7 $$\begin{aligned}&a \left( \eta _{\phi ,\psi }+\eta _{\zeta ,\theta }\right) -6 \chi f_{,\psi } \eta _{a,\theta }=0, \end{aligned}$$

A8 $$\begin{aligned}&a \left( \eta _{\phi ,\zeta }+\eta _{\psi ,\theta }\right) +6 \eta _{a,\theta }=0, \end{aligned}$$

A9 $$\begin{aligned}&6 \chi f_{,\phi } \eta _{a,\phi } -a \eta _{\theta ,\phi } =0,\end{aligned}$$

A10 $$\begin{aligned}&6 \chi f_{,\psi }\eta _{a,\psi }-a\eta _{\zeta ,\psi }=0,\end{aligned}$$

A11 $$\begin{aligned}&6 \eta _{a,\zeta }+a \eta _{\psi ,\zeta }=0,\end{aligned}$$

A12 $$\begin{aligned}&h_{,a}-6 a^2 \left( \chi f_{,\phi } \eta _{\phi ,t} +\chi f_{,\psi } \eta _{\psi ,t} -\eta _{\zeta ,t}\right) \nonumber \\&\quad +\,12 a (\xi f-\theta -1) \eta _{a,t}=0, \end{aligned}$$

A13 $$\begin{aligned}&6 a\left( \chi f_{,\psi } \eta _{\psi ,\theta }-\eta _{\zeta ,\theta }\right) \nonumber \\&\quad +\,12 (\theta +1-\xi f) \eta _{a,\theta }-a^2 \eta _{\phi ,a}=0,\end{aligned}$$

A14 $$\begin{aligned}&6 \chi (f_{,\phi } \eta _{a,\psi }+ f_{,\psi } \eta _{a,\phi })-a \left( \eta _{\theta ,\psi }+\eta _{\zeta ,\phi }\right) =0, \end{aligned}$$

A15 $$\begin{aligned}&6 (\chi f_{,\phi }\eta _{a,\zeta }-\eta _{a,\phi })-a \left( \eta _{\psi ,\phi }+\eta _{\theta ,\zeta }\right) =0,\end{aligned}$$

A16 $$\begin{aligned}&6 \chi f_{,\phi } \eta _{a,\theta }-a \left( \eta _{\phi ,\phi }+\eta _{\theta ,\theta }-\lambda _{,t}\right) -3\eta _{a}=0,\end{aligned}$$

A17 $$\begin{aligned}&6 \chi f_{,\psi }\eta _{a,\zeta } -6\eta _{a,\psi }-3 \eta _{a}\nonumber \\&\quad -\,a \left( \eta _{\psi ,\psi }+\eta _{\zeta ,\zeta }-\lambda _{,t}\right) =0,\end{aligned}$$

A18 $$\begin{aligned}&6 a \left( \chi f_{,\phi } \eta _{\phi ,\phi }+\chi f_{,\phi \phi } \eta _{\phi }+\chi f_{,\phi }\eta _{a,a} +\chi f_{,\psi }\eta _{\psi ,\phi }\right. \nonumber \\&\quad \left. +\,\chi f_{,\phi \psi }\eta _{\psi }-\chi \lambda _{,t}f_{,\phi }-\eta _{\zeta ,\phi }\right) +12 \chi f_{,\phi }\eta _{a}\nonumber \\&\quad +\,12 (\theta +1-\xi f) \eta _{a,\phi }\nonumber \\&\quad -\,a^2 \eta _{\theta ,a}=0, \end{aligned}$$

A19 $$\begin{aligned}&6 a \left( \chi f_{,\phi } \eta _{\phi ,\zeta } +\,\chi f_{,\psi } \eta _{\psi ,\zeta }-\eta _{a,a}-\eta _{\zeta ,\zeta } +\lambda _{t}\right) \nonumber \\&\quad +\,12 (\theta +1-\xi f) \eta _{a,\zeta }\nonumber \\&\quad -\,12 \eta _{a} -a^2 \eta _{\psi ,a}=0, \end{aligned}$$

A20 $$\begin{aligned}&6 a \left( \chi f_{,\phi }\eta _{\phi ,\psi } +\,\chi f_{,\phi \psi }\eta _{\phi }+\chi f_{,\psi } \left( \eta _{a,a}+\eta _{\psi ,\psi }-\lambda _{t}\right) \right. \nonumber \\&\quad \left. +\,\chi f_{,\psi \psi } \eta _{\psi } -\eta _{\zeta ,\psi }\right) \nonumber \\&\quad +\,12 \chi f_{,\psi }\eta _{a}+12 (\theta +1-\xi f) \eta _{a,\psi }\nonumber \\&\quad -\,a^2 \eta _{\zeta ,a}=0, \end{aligned}$$

A21 $$\begin{aligned}&a \left( \chi a f_{,\phi }\eta _{\phi ,a} -\,\xi f_{,\phi }\eta _{\phi }+\chi a f_{,\psi }\eta _{\psi ,a}\nonumber \right. \\&\quad -\,\xi f_{,\psi }\eta _{\psi }-2 \xi f \eta _{a,a}+\xi \lambda _{,t}f+2 \theta \eta _{a,a}\nonumber \\&\quad \left. +\,2 \eta _{a,a}+\eta _{\theta }-a \eta _{\zeta ,a}- (\theta +1)\lambda _{t}\right) \nonumber \\&\quad +\,(\theta +1-\xi f)\eta _{a}=0, \end{aligned}$$

where commas denote partial derivatives. The solutions of these equations are the functions (A2). Clearly, being a system of non-linear partial differential equations, the solution is not unique. This means that several Noether symmetries can be selected according to the different functions (A2).
